# Development and validation of an interpretable predictive model for mismatch between endoscopic ultrasonography and histopathological diagnosis in colorectal subepithelial lesions: a retrospective single-center study

**DOI:** 10.3389/fmed.2026.1831811

**Published:** 2026-07-30

**Authors:** Run-hua Li, Xiao-hua Bao, Kai-yun Liu, Wen Xu, Si-cun Lu, Lei Wang, Bingfeng He, Ying Zhu, Wei Gong, Xiaobing Cui

**Affiliations:** 1 Department of Gastroenterology, Shenzhen Hospital, Southern Medical University, Shenzhen, Guangdong, China; 2 Shenzhen School of Clinical Medicine, Southern Medical University, Shenzhen, Guangdong, China; 3 Department of Gastroenterology, No. 964th Hospital, Changchun, Jilin, China; 4 Department of Clinical Medicine, Hangzhou Medical College, Hangzhou, Zhejiang, China; 5 Wuzhou Medical College, Wuzhou, Guangxi, China

**Keywords:** colorectal subepithelial lesions, diagnostic mismatch, endoscopic ultrasonography, histopathology, machine learning, SHapley Additive exPlanations (SHAP)

## Abstract

**Background:**

Endoscopic ultrasonography (EUS) is important for evaluating colorectal subepithelial lesions (SELs), but EUS–histopathology mismatch may complicate preoperative management. Evidence for predicting mismatch risk in colorectal SELs remains limited.

**Methods:**

We retrospectively included patients with colorectal SELs who underwent EUS and had histopathological confirmation. Candidate predictors were selected using LASSO, and seven machine-learning algorithms were developed and compared. Performance was assessed by discrimination, calibration, decision curve analysis, and repeated stratified 10-fold cross-validation. SHAP was used for model interpretation, and the final model was deployed as a web-based calculator.

**Results:**

Among 301 SELs, 222 were concordant and 79 showed mismatch (26.2%). LASSO selected lesion location, echogenicity, and EUS layer of origin. The SVM model achieved the best overall test-set performance, with an AUC of 0.818 (95% CI, 0.673–0.964), accuracy of 0.883, sensitivity of 0.538, specificity of 0.979, PPV of 0.875, NPV of 0.885, F1-score of 0.667, and Brier score of 0.109. Repeated cross-validation yielded an SVM AUC of 0.713 (95% interval, 0.537–0.884). SHAP identified lesion location as the strongest contributor to mismatch risk, followed by echogenicity and layer of origin.

**Conclusion:**

An interpretable SVM model based on routinely available EUS features may help identify colorectal SELs at higher risk of EUS–histopathology mismatch and support post-EUS risk alerting. Prospective multicenter validation is required before broader clinical implementation.

## Introduction

Colorectal subepithelial lesions (SELs) are mass-like structures arising from the submucosal layer within the gastrointestinal (GI) lumen and are typically covered by normal-appearing epithelium (1, 2). Owing to substantial heterogeneity in biological behavior, the pathological spectrum of SELs ranges from benign leiomyomas to potentially aggressive entities such as gastrointestinal stromal tumors (GISTs) and neuroendocrine tumors (NETs) (3). Therefore, accurate endoscopic diagnosis is essential for guiding appropriate management and avoiding both overtreatment and delayed intervention (4, 5).

Compared with upper GI SELs, colorectal SELs are relatively uncommon (4) and show greater diversity in pathological composition and imaging features. In addition, the colorectum is anatomically narrower and more tortuous, making EUS manipulation and acquisition of stable images more challenging. Clinical decision-making in this setting often requires balancing the assessment of malignant potential against the need for tissue acquisition or resection. Previous studies have reported an overall diagnostic accuracy of EUS for SELs ranging from approximately 45.5% to 66.7% (6–8). In colorectal SELs, diagnostic accuracy may vary even more widely because of lesion rarity, heterogeneous pathology, and procedure-related constraints.

Endoscopic ultrasonography (EUS) is regarded as a first-line modality for evaluating SELs. It provides key information on the layer of origin, lesion size, echogenic pattern, internal heterogeneity, margins, and vascularity, and therefore plays an important role in preoperative differential diagnosis and risk assessment (9, 10). However, EUS-based diagnosis remains challenging because diagnostic criteria are complex, lesions often share similar endoscopic appearances (5), and operator experience varies, with limited interobserver agreement (11, 12). Consequently, the diagnostic performance of EUS for colorectal SELs remains suboptimal, and prior studies have reported diagnostic accuracy as low as 48% in this setting (6).

In clinical practice, EUS–histopathology mismatch, defined as discordance between EUS-based and histopathological diagnoses, is not uncommon. Such mismatch may lead to underestimation of higher-risk lesions with delayed treatment, or overestimation of benign lesions with unnecessary intervention, increased healthcare costs, and procedure-related risks. Improving overall diagnostic accuracy alone may not be sufficient for individualized decision-making. Identifying cases with low diagnostic reliability on EUS before definitive treatment could help recognize patients at higher risk of EUS–histopathology mismatch, guide subsequent diagnostic and therapeutic strategies, optimize resource allocation, and reduce avoidable risks.

To date, studies of EUS diagnostic discrepancies in colorectal SELs have mainly focused on overall accuracy assessment or on identifying associated factors using conventional statistical models (13, 14). However, traditional regression approaches rely on prespecified linear relationships and may not fully capture nonlinear effects or complex feature interactions that are common in clinical data. Their robustness in feature selection and individualized risk estimation may also be limited in settings with multiple predictors, collinearity, or high-dimensional information. In colorectal SELs, interactions between anatomical location and EUS characteristics may further complicate stable model development using purely linear methods. Moreover, the lack of interpretable and deployable tools has limited clinical translation.

Machine-learning methods have shown potential in medical predictive modeling by capturing complex patterns in multidimensional clinical data. Nevertheless, their “black-box” nature remains a barrier to clinical interpretation and implementation. SHapley Additive exPlanations (SHAP), a model-agnostic interpretability method, can quantify the marginal contribution of each feature to model predictions and reveal feature directionality and nonlinear relationships. Thus, SHAP may enhance model transparency and clinical interpretability while preserving predictive performance.

Accordingly, using a retrospective cohort of colorectal SELs, we developed and validated an interpretable machine-learning model to predict the risk of EUS–histopathology mismatch. This model aimed to provide explainable preoperative decision support, facilitate individualized risk stratification, and assist clinicians in identifying colorectal SELs for which EUS-based diagnosis may require further verification.

## Materials and methods

### Study design and participants

**FIGURE 1:**
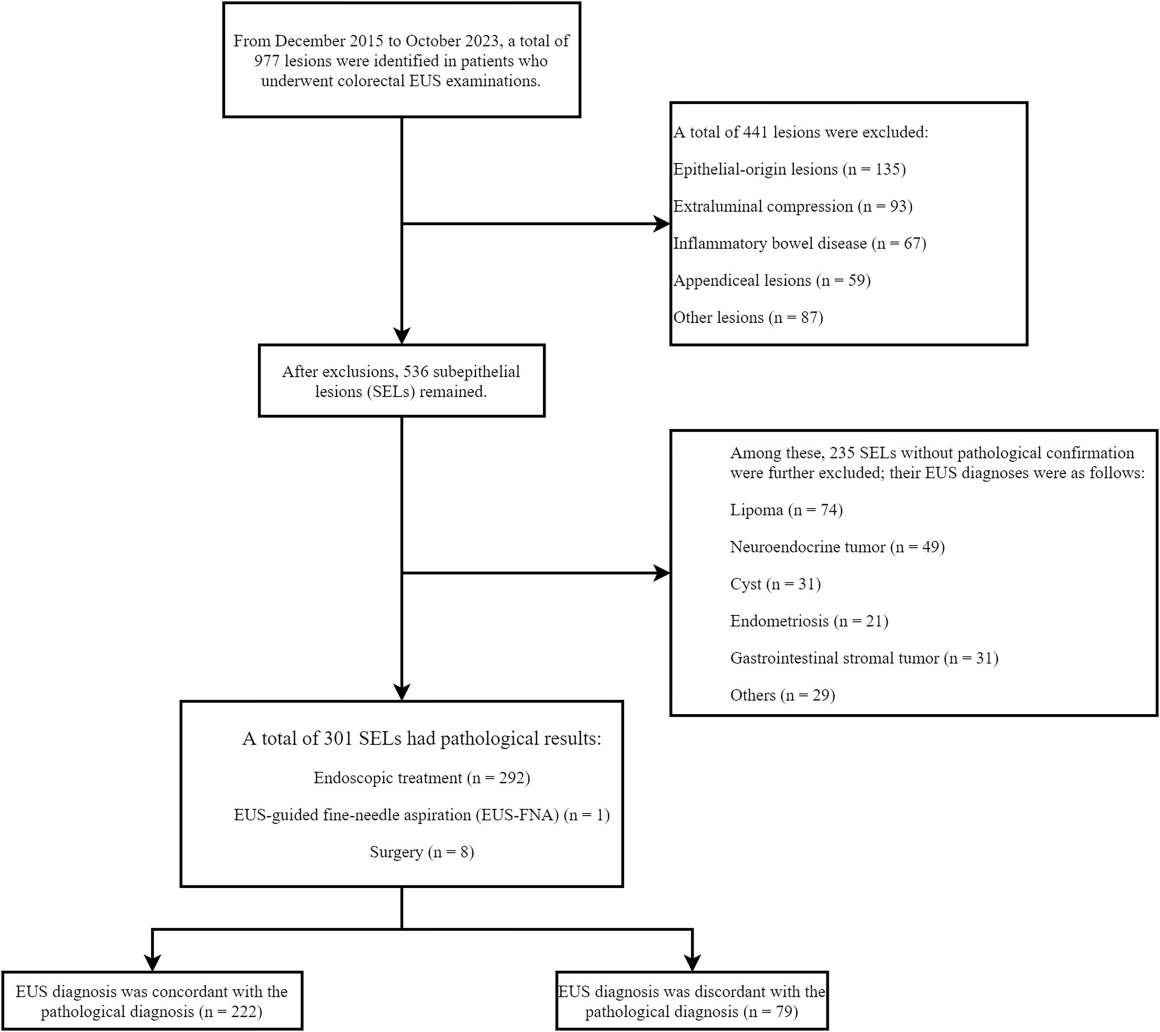
Flow diagram of patient/lesion selection.

This retrospective, single-center cohort study was conducted at Shenzhen Hospital of Southern Medical University. We screened patients who were suspected of having colorectal subepithelial lesions (SELs) on colonoscopy and subsequently underwent endoscopic ultrasonography (EUS) between December 2015 and October 2023. Lesions were used as the unit of analysis, and only one colorectal SEL was included per patient. In accordance with the Chinese Consensus on Endoscopic Diagnosis and Management of Gastrointestinal Submucosal Tumor (Version 2018) (15), histopathological diagnosis was established by endoscopic resection, EUS-guided fine-needle aspiration (EUS-FNA), or surgical resection. Only lesions with definitive histopathological results were included. The patient selection flowchart is shown in Figure 1.

The inclusion criteria were as follows: (1) colorectal SEL confirmed by EUS; and (2) complete clinical data. The exclusion criteria were as follows: (1) lesions considered epithelial in origin on EUS; (2) lesions considered appendiceal in origin on EUS; (3) lesions considered perianal in origin on EUS; (4) patients deemed unsuitable for inclusion by the investigators; and (5) missing key variables or histopathological results.

This study was conducted in accordance with the Declaration of Helsinki and was approved by the Ethics Committee of Shenzhen Hospital of Southern Medical University (NYSZYYEC20230088). The study was retrospectively registered (NCT07351890). Written informed consent was obtained from all patients or their legal representatives.

### Selection of predictors

This study aimed to identify key determinants of discordance between EUS-based assessment and histopathological findings and to develop an individualized risk prediction model for EUS–histopathology mismatch. EUS–histopathology mismatch was defined as discordance between the EUS-based diagnosis of the lesion’s pathological subtype and the final histopathological diagnostic category, namely disagreement at the specific pathological type level. For ambiguous or non-definitive EUS diagnoses, such as “suspected,” “possible,” or “probable” diagnoses, the primary EUS diagnostic category was used for classification. For example, “suspected GIST” was categorized as GIST at the EUS level and was considered concordant only when the final histopathological diagnosis was GIST. When multiple differential diagnoses were listed, only the primary EUS diagnosis was used to determine concordance; secondary or alternative diagnoses were not counted as matches. No partial-match category was used.

Candidate predictors were extracted from routinely collected clinical records and structured EUS reports and included demographic characteristics, lesion imaging features, and operator-related factors. The variables included sex, age, lesion location, maximum lesion diameter, echogenicity, EUS layer of origin, image quality, physician type, pre-/post-training status, and EUS modality. EUS–histopathology mismatch was treated as the positive class, and concordance was treated as the negative class. Detailed variable definitions are provided in Supplementary Table 1.

### Data preprocessing

Data preprocessing was performed in R. To ensure consistency across the statistical and modeling workflows, categorical variables were converted into factors, whereas continuous variables were retained as numeric variables. For models that rely on distance metrics or are sensitive to feature scaling, the input feature matrix was standardized. All scaling parameters were estimated exclusively from the training set and then applied to the test set to prevent information leakage. For models requiring one-hot encoding, model.matrix() was used to transform factor variables into dummy variables. Feature matrices for the training and test sets were harmonized to ensure identical dimensionality and column order, thereby avoiding mismatches caused by differences in factor levels.

Regarding missing data, all candidate predictors for the 301 included lesions were derived from mandatory fields in the institution’s structured EUS reporting template and electronic medical record system. Data completeness was further verified through a second round of investigator review and quality control before export. Because the missingness rate was 0%, no imputation was performed, and no samples were excluded because of missing data.

### Data splitting and feature selection

The dataset was randomly divided into a training set and a test set at an 80:20 ratio. The training set included 241 lesions, and the test set included 60 lesions. Model training, hyperparameter tuning, and threshold determination were performed exclusively in the training set, whereas the test set was reserved for independent validation to minimize information leakage and reduce the risk of overfitting.

Feature selection was performed in the training set using least absolute shrinkage and selection operator (LASSO) logistic regression. LASSO introduces an L1 penalty that shrinks some regression coefficients to zero, thereby enabling automated variable selection. The penalty parameter λ was selected using 10-fold cross-validation, and the more parsimonious λ1se value was used as the final parameter. The features selected by LASSO were then fixed and consistently used to develop all subsequent machine-learning models, thereby avoiding feature-selection bias and information leakage.

### Predictive models

We developed and compared seven binary machine-learning models to predict the risk of EUS–histopathology mismatch: logistic regression, k-nearest neighbors (KNN), decision tree, random forest, support vector machine (SVM), neural network, and XGBoost (eXtreme Gradient Boosting).

Hyperparameters for each model were optimized within the training set using cross-validation and/or grid search. To address class imbalance, class weights were applied in the SVM model to improve recognition of the positive class.

### Probability output and calibration

To enable calibration assessment, including calibration curves and Brier score calculation, as well as decision curve analysis (DCA), predicted probabilities were used as the unified model outputs. For models that natively provide probabilistic predictions, such as logistic regression, random forest, and XGBoost, predicted probabilities were directly extracted. For models whose native outputs are not probabilities, such as SVM, probability estimation and calibration were performed in the training set based on model outputs to transform them into interpretable probability values. All parameters related to probability estimation and calibration were determined exclusively within the training set and then fixed before application to the test set, thereby avoiding information leakage and optimistic bias.

### Model evaluation

Model performance was assessed in four domains: discrimination, threshold-dependent metrics, calibration, and clinical net benefit. (1) Discrimination: Receiver operating characteristic (ROC) curves were generated, and the area under the ROC curve (AUC) was calculated. For the test set, 95% confidence intervals (CIs) for the AUC were estimated using the DeLong method. To address uncertainty associated with the relatively small hold-out test set, we additionally performed repeated stratified 10-fold cross-validation as a robustness analysis. The cross-validation procedure was repeated 10 times, yielding 100 validation folds. In each resampling procedure, model training and hyperparameter tuning were conducted within the training folds, and AUC was calculated in the corresponding validation fold. Cross-validated AUCs were summarized with empirical 95% intervals across repeated validation folds. This analysis was intended to assess the robustness of overall discriminatory performance and did not replace the predefined hold-out test-set evaluation.(2) Threshold-dependent metrics: Accuracy, sensitivity, specificity, positive predictive value (PPV), negative predictive value (NPV), and F1-score were reported.

(3) Threshold selection: To avoid information leakage from the test set, we adopted a “threshold selection in the training set and validation in the test set” strategy. For each model, the optimal threshold was determined in the training set by maximizing the Youden index, defined as sensitivity + specificity - 1. This threshold was then fixed and applied to the test set for performance evaluation. (4) Calibration: Calibration curves and the Brier score were used to evaluate agreement between predicted probabilities and observed event rates.(5) Clinical net benefit: DCA was performed to compare the net benefit of different models across a range of threshold probabilities and to assess their potential clinical utility.

### Model interpretability

Because machine-learning models often lack intuitive interpretability, we applied SHapley Additive exPlanations (SHAP) to enhance model transparency and clinical usability. Grounded in game theory, SHAP quantifies the marginal contribution of each feature to the predicted outcome and provides both global- and individual-level explanations. Global SHAP analysis was used to summarize overall feature importance and depict the direction and pattern of each feature’s effect on EUS–histopathology mismatch risk. Local SHAP analysis was used to explain how a specific patient’s feature profile contributed to the individualized predicted risk. By integrating global and local interpretability, SHAP helped mitigate the “black-box” nature of machine-learning models and improved the clinical interpretability of the final model.

### Online prediction platform

To facilitate clinical implementation, we developed a web-based prediction platform using R Shiny. Clinicians can input relevant patient-level features to obtain an individualized prediction of EUS–histopathology mismatch risk and view patient-level SHAP contributions ranked by importance. This platform provides real-time insight into the key factors driving the estimated risk for each case.

### Statistical analysis

Continuous variables are presented as mean ± standard deviation (SD) or median with interquartile range (IQR), as appropriate. Categorical variables are presented as counts and percentages. Between-group comparisons were performed using Student’s *t-*test or the Mann–Whitney U test for continuous variables, depending on data distribution, and the chi-square test or Fisher’s exact test for categorical variables.

Statistical inference for model performance was conducted as follows: the 95% CI for the test-set AUC was calculated using the DeLong method; and the 95% CIs for accuracy, sensitivity, specificity, PPV, and NPV were estimated using the exact binomial, or Clopper–Pearson, method. All tests were two-sided, and *p* < 0.05 was considered statistically significant.

## Results

### Patient selection and pathological confirmation

Between December 2015 and October 2023, a total of 977 colorectal EUS examinations were recorded. Among these, 441 non-SEL-related lesions were excluded, including epithelial-origin lesions (*n* = 135), extraluminal compression (*n* = 93), inflammatory bowel disease (*n* = 67), appendiceal lesions (*n* = 59), and other lesions (*n* = 87). Finally, 536 colorectal SELs were identified.

Because the primary outcome was concordance between EUS-based and histopathological diagnoses, with histopathology serving as the reference standard, 235 SELs without pathological confirmation were further excluded. The main EUS-based diagnoses among these non-biopsied or non-resected lesions were lipoma (*n* = 74), neuroendocrine tumor (*n* = 49), cyst (*n* = 31), endometriosis (*n* = 21), gastrointestinal stromal tumor (GIST) (*n* = 31), and others (*n* = 29). In total, 301 SELs with histopathological confirmation were included for model development. Histopathology was obtained by endoscopic treatment in 292 lesions, EUS-guided fine-needle aspiration in 1 lesion, and surgery in 8 lesions. Among the 301 included SELs, EUS and histopathological diagnoses were concordant in 222 lesions and discordant in 79 lesions (Figure 1).

### Baseline clinical characteristics

A total of 301 colorectal SELs were included, of which 222 lesions were classified as concordant between EUS and histopathology (match group, 73.8%) and 79 lesions were classified as EUS–histopathology mismatch (mismatch group, 26.2%). The mean age of the overall cohort was 48.13 ± 11.80 years, and 178 patients (59.1%) were male. No significant differences were observed between the match and mismatch groups in age, sex, or maximum lesion diameter.

Lesion location differed significantly between the two groups (*P* < 0.001). Compared with the match group, the mismatch group had a lower proportion of rectal lesions (50.6% vs. 79.7%) and higher proportions of left-colon lesions (22.8% vs. 5.4%) and right-colon lesions (21.5% vs. 11.7%). EUS layer of origin also differed significantly between groups (*P* = 0.001): layer-4 lesions were more frequent in the mismatch group (10.1% vs. 1.4%), whereas layer-3 lesions were less frequent (50.6% vs. 64.9%). Echogenicity showed a non-significant trend toward between-group differences (*P* = 0.068).

Among operator- and examination-related variables, training status differed significantly between groups (*P* = 0.018), with a higher proportion of pre-training cases in the mismatch group (53.2% vs. 37.8%). Image quality, physician type, and EUS modality did not differ significantly between groups. Overall, lesion location, EUS layer of origin, and training status were associated with EUS–histopathology mismatch in baseline comparisons, whereas age, lesion size, sex, echogenicity, image quality, physician type, and EUS modality were not significantly different. Detailed baseline characteristics are shown in Table 1.

**TABLE 1:** Baseline characteristics of participants in this study.

Variables	Total (*n* = 301)	Match (*n* = 222)	Mismatch(*n* = 79)	Statistic	*P*
Age, mean ± SD	48.13 ± 11.80	48.14 ± 12.10	48.10 ± 10.99	*t* = 0.03	0.978
Size Mm, mean ± SD	6.44 ± 3.43	6.50 ± 3.60	6.25 ± 2.94	*t* = 0.56	0.578
Sex, *n* (%)				χ^2^ = 0.525	0.469
Female	123 (40.864)	88 (39.640)	35 (44.304)		
Male	178 (59.136)	134 (60.360)	44 (55.696)		
Location, *n* (%)				χ^2^ = 29.005	<0.001
Left colon	30 (9.967)	12 (5.405)	18 (22.785)		
Rectum	217 (72.093)	177 (79.730)	40 (50.633)		
Right colon	43 (14.286)	26 (11.712)	17 (21.519)		
Terminal ileum	11 (3.654)	7 (3.153)	4 (5.063)		
Echo, *n* (%)				χ^2^ = 5.364	0.068
Anechoic	12 (3.987)	11 (4.955)	1 (1.266)		
Hyperechoic	44 (14.618)	37 (16.667)	7 (8.861)		
Hypoechoic	245 (81.395)	174 (78.378)	71 (89.873)		
Eus Layer, *n* (%)				χ^2^ = 16.221	0.001
Layer 2	52 (17.276)	34 (15.315)	18 (22.785)		
Layer 2 + 3	54 (17.940)	41 (18.468)	13 (16.456)		
Layer 3	184 (61.130)	144 (64.865)	40 (50.633)		
Layer 4	11 (3.654)	3 (1.351)	8 (10.127)		
Img Q, *n* (%)				χ^2^ = 5.564	0.062
High	145 (48.173)	107 (48.198)	38 (48.101)		
Low	46 (15.282)	28 (12.613)	18 (22.785)		
Medium	110 (36.545)	87 (39.189)	23 (29.114)		
Doc type, *n* (%)				χ^2^ = 1.934	0.164
Junior	95 (31.561)	75 (33.784)	20 (25.316)		
Senior	206 (68.439)	147 (66.216)	59 (74.684)		
Training, *n* (%)				χ^2^ = 5.624	0.018
Post-training	175 (58.140)	138 (62.162)	37 (46.835)		
Pre-training	126 (41.860)	84 (37.838)	42 (53.165)		
Eus Type, *n* (%)				–	0.092
Linear	6 (1.993)	6 (2.703)	0 (0.00)		
Mini-probe	69 (22.924)	56 (25.225)	13 (16.456)		
Radial	226 (75.083)	160 (72.072)	66 (83.544)		

t, *t*-test, Z, Mann-Whitney test, χ^2^, Chi-square test, –, Fisher exact. SD, standard deviation; M, median; Q1, 1st quartile; Q3, 3rd quartile.

### Pathological spectrum and primary EUS diagnostic spectrum

As shown in Table 2, neuroendocrine tumors accounted for more than half of the final histopathological diagnoses among the 301 colorectal SELs, followed by common benign lesions such as lipomas and leiomyomas. Other pathological entities, including rectal tonsil, chronic mucosal inflammation, granular cell tumor, cysts, fibrous hyperplasia, and other rare lesions, were also observed, indicating substantial pathological heterogeneity in this cohort.

**TABLE 2:** Spectrum of final histopathological diagnoses and primary EUS diagnoses (*n* = 301).

Final histopathological diagnosis	*n*	%	Primary EUS diagnosis	*n*	%
Neuroendocrine tumor	169	56.1%	Neuroendocrine tumor	229	76.1%
Lipoma	35	11.6%	Lipoma	35	11.6%
Leiomyoma	17	5.6%	Cyst	10	3.3%
Rectal tonsil	13	4.3%	Gastrointestinal stromal tumor (GIST)	9	3.0%
Chronic mucosal inflammation	12	4.0%	Leiomyoma	4	1.3%
Granular cell tumor	10	3.3%	Intestinal schistosomiasis	4	1.3%
Cyst	9	3.0%	Fibrous hyperplasia	3	1.0%
Fibrous hyperplasia	7	2.3%	Lymphangioma	2	0.7%
Others	29	9.6%	Others	5	1.7%

EUS, endoscopic ultrasonography.

In contrast, the distribution of primary EUS diagnoses was more concentrated. Neuroendocrine tumor was the most frequent primary EUS diagnosis, with a proportion higher than that observed in final histopathology. Lipoma and cyst were also commonly diagnosed on EUS, whereas other diagnostic categories accounted for relatively small proportions.

Endoscopic ultrasonography–histopathology concordance varied markedly across pathological subtypes. As shown in Supplementary Table 2, concordance was relatively high for neuroendocrine tumors, cysts, and lipomas, whereas lower match rates were observed for chronic mucosal inflammation, leiomyoma, granular cell tumor, rectal tonsil, and the “Others” category.

### Clinical scenario–stratified concordance analysis

To further characterize the clinical contexts in which EUS–histopathology mismatch occurred, we examined concordance rates across predefined clinical and EUS-related scenarios before model development. Detailed results are provided in Supplementary Table 3.

Concordance varied substantially according to lesion location. Rectal lesions showed the highest concordance rate, with 177 of 217 lesions concordant with histopathology (81.6%). In contrast, concordance was lower for lesions located in the terminal ileum (7/11, 63.6%), right colon (26/43, 60.5%), and left colon (12/30, 40.0%). These findings suggest that lesions outside the rectum, particularly those in the left colon, are more likely to be associated with EUS–histopathology mismatch.

Concordance also differed according to echogenicity and EUS layer of origin. Anechoic lesions had the highest concordance rate (11/12, 91.7%), followed by hyperechoic lesions (37/44, 84.1%), whereas hypoechoic lesions showed a lower concordance rate (174/245, 71.0%). Regarding EUS layer of origin, layer-3 lesions showed a concordance rate of 78.3% (144/184), whereas layer-4 lesions had the lowest concordance rate (3/11, 27.3%). These results indicate that mismatch was more frequent in scenarios characterized by non-specific hypoechoic appearance or deeper-layer involvement.

Operator- and examination-related scenarios were also explored. Post-training examinations showed a higher concordance rate than pre-training examinations (138/175, 78.9% vs. 84/126, 66.7%). Although the crude concordance rate was numerically lower among senior physicians than among junior physicians (147/206, 71.4% vs. 75/95, 78.9%), physician type was not significantly associated with mismatch in the baseline comparison. Image quality and EUS modality also showed varying concordance patterns, but these variables were not retained in subsequent LASSO-based feature selection.

Overall, these scenario-stratified findings indicate that EUS–histopathology mismatch was not randomly distributed, but was closely related to lesion location, echogenicity, and EUS layer of origin. This clinical pattern provided the rationale for subsequent feature selection and predictive modeling.

### Feature selection

In the training set, feature selection was performed using least absolute shrinkage and selection operator (LASSO) logistic regression to reduce multicollinearity and obtain a parsimonious set of predictors. LASSO applies an L1 penalty that shrinks some coefficients to zero, thereby enabling automated variable selection.

**FIGURE 2:**
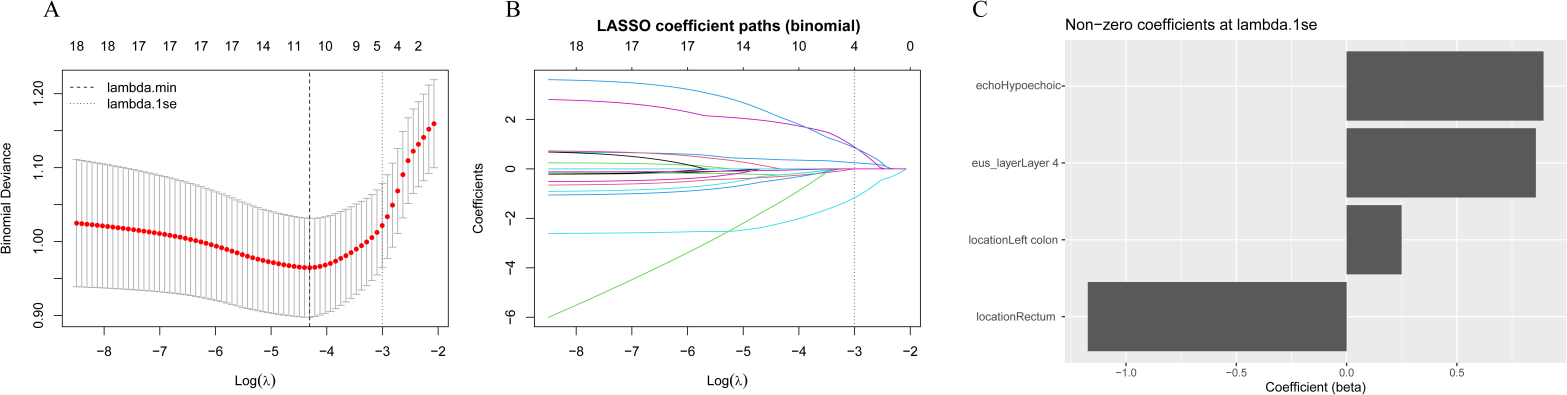
Feature selection in the training set using LASSO regression. **(A)** Ten-fold cross-validation was used to select the penalty parameter (λ); dashed vertical lines indicate λ_min and λ_1se, and the more robust λ_1se was used to determine the final feature set.**(B)** LASSO coefficient paths for candidate predictors: as λ increases (stronger penalization), coefficients progressively shrink toward zero, reflecting the variable selection process.**(C)** Predictors with non-zero coefficients at λ_1se. Three variables were ultimately retained: lesion location (location), echogenicity (echo), and EUS layer of origin (eus_layer). LASSO, least absolute shrinkage and selection operator; EUS, endoscopic ultrasonography; SELs, subepithelial lesions.

As shown in Figure 2A, the penalty parameter was determined using 10-fold cross-validation, and the more parsimonious penalization level was selected as the final λ. Figure 2B shows the coefficient shrinkage trajectories of candidate variables across different penalty strengths, illustrating the transition from a complex model to a sparse solution. Variables with non-zero coefficients under the final λ are shown in Figure 2C.

Least absolute shrinkage and selection operator selected three features: lesion location, echogenicity, and EUS layer of origin. These variables were subsequently used to develop all downstream machine-learning models for predicting EUS–histopathology mismatch risk.

### Hyperparameter tuning

All hyperparameter tuning was performed exclusively within the training set using cross-validation and/or grid search, with cross-validated AUC as the unified optimization metric. The test set was reserved solely for final independent performance evaluation to prevent information leakage and reduce the risk of overfitting.

Given differences in model structure and computational complexity, candidate hyperparameter spaces were systematically searched using either 5-fold or 10-fold cross-validation in the training set. Final hyperparameters were selected according to the best internal cross-validated AUC. For models without tunable hyperparameters, such as logistic regression, a standard binomial logit model was fitted. For all other models, structural or tuning parameters were optimized within the training set.

The optimal hyperparameters and tuning strategies for each model are summarized in Supplementary Table 4. Based on these settings, all models were trained in the training set and then evaluated in the independent test set.

### Model performance comparison

Based on the three LASSO-selected predictors, seven machine-learning models were developed and compared, including logistic regression, KNN, decision tree, random forest, SVM, neural network, and XGBoost. For all models, classification thresholds were determined in the training set by maximizing the Youden index and were then fixed for test-set evaluation.

Model performance was evaluated across discrimination, threshold-dependent classification metrics, calibration, and clinical net benefit. F1-score was used to summarize positive-class performance under class imbalance, and the Brier score was used to quantify the discrepancy between predicted probabilities and observed outcomes.

**FIGURE 3:**
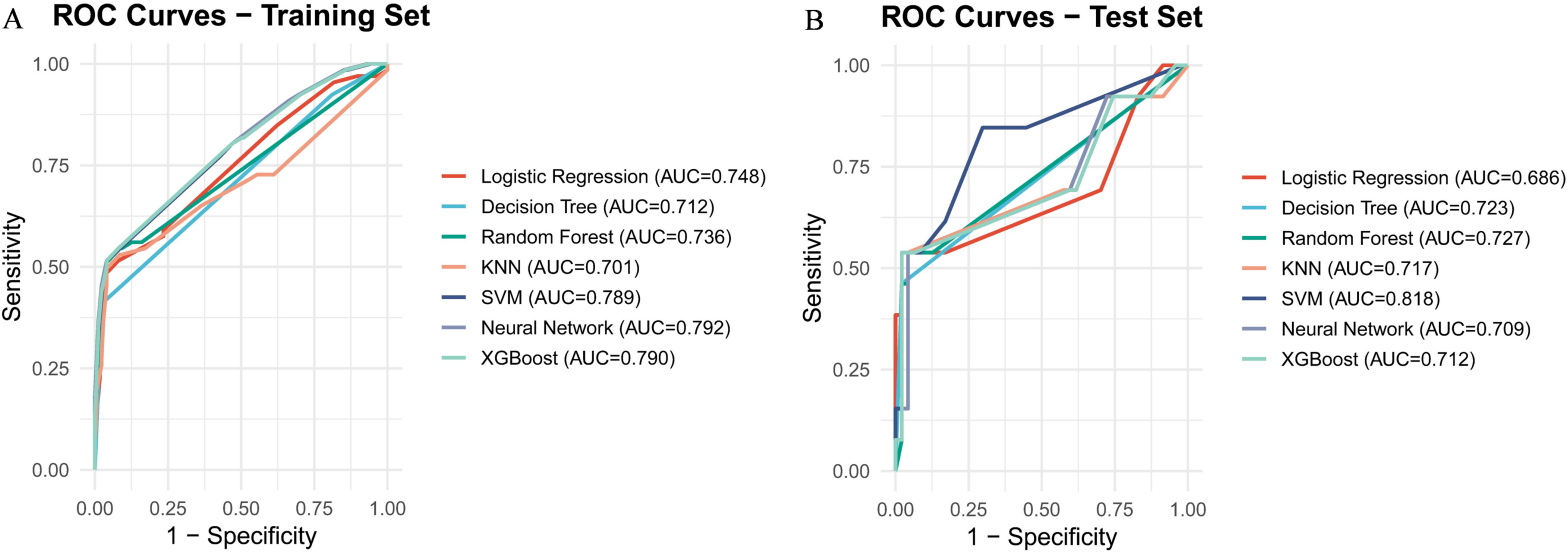
Receiver operating characteristic (ROC) curves of seven machine learning models in the training set **(A)** and testing set **(B)**.

In the training set, AUCs ranged from 0.701 to 0.792 (Figure 3A and Supplementary Table 5). Although some more complex models showed slightly higher AUCs in the training set, this advantage did not consistently persist in the independent test set. In contrast, SVM achieved the highest test-set AUC of 0.818 (95% CI, 0.673–0.964) (Figure 3B and Table 3), indicating favorable generalization.

**TABLE 3:** Performance of each algorithm in the testing set.

Model	AUC (95% CI)	Accuracy (95% CI)	Sensitivity (95% CI)	Specificity (95% CI)	PPV (95% CI)	NPV (95% CI)	F1-score	Brier score	Threshold_Youden (determined by the training set)
KNN	0.717 (0.526–0.907)	0.867 (0.754–0.941)	0.538 (0.251–0.808)	0.957 (0.855–0.995)	0.778 (0.400–0.972)	0.882 (0.761–0.956)	0.636	0.125	0.350
Random forest	0.727 (0.570–0.885)	0.867 (0.754–0.941)	0.538 (0.251–0.808)	0.957 (0.855–0.995)	0.778 (0.400–0.972)	0.882 (0.761–0.956)	0.636	0.127	0.322
Neural network	0.709 (0.523–0.894)	0.883 (0.774–0.952)	0.538 (0.251–0.808)	0.979 (0.887–0.999)	0.875 (0.473–0.997)	0.882 (0.766–0.956)	0.636	0.128	0.395
Logistic regression	0.686 (0.481–0.890)	0.883 (0.774–0.952)	0.538 (0.251–0.808)	0.979 (0.887–0.999)	0.875 (0.473–0.997)	0.885 (0.766–0.956)	0.667	0.110	0.256
Decision tree	0.723 (0.562–0.883)	0.867 (0.754–0.941)	0.462 (0.192–0.749)	0.979 (0.887–0.999)	0.857 (0.421–0.996)	0.868 (0.747–0.945)	0.600	0.119	0.534
XGBoost	0.712 (0.522–0.902)	0.867 (0.754–0.941)	0.538 (0.251–0.808)	0.957 (0.855–0.995)	0.778 (0.400–0.972)	0.882 (0.761–0.956)	0.636	0.118	0.318
SVM	0.818 (0.673–0.964)	0.883 (0.774–0.952)	0.538 (0.251–0.808)	0.979 (0.887–0.999)	0.875 (0.473–0.997)	0.885 (0.766–0.956)	0.667	0.109	0.485

AUC, area under the receiver operating characteristic curve; CI, confidence interval; PPV, positive predictive value; NPV, negative predictive value; KNN, k-nearest neighbors; XGBoost, eXtreme Gradient Boosting; SVM, support vector machine.

In the test set, sensitivities were broadly comparable across models, whereas greater differences were observed in specificity, accuracy, and positive predictive value (PPV). SVM achieved the highest overall accuracy (0.883; 95% CI, 0.774–0.952) and specificity (0.979; 95% CI, 0.887–0.999), suggesting an advantage in reducing false-positive predictions (Table 3). SVM also yielded a high PPV of 0.875 (95% CI, 0.473–0.997), indicating greater reliability when predicting mismatch. Its negative predictive value (NPV) was 0.885 (95% CI, 0.766–0.956), comparable to that of other models and indicating that improved positive predictive performance was not achieved at the expense of reduced reliability for negative predictions.

**FIGURE 4:**
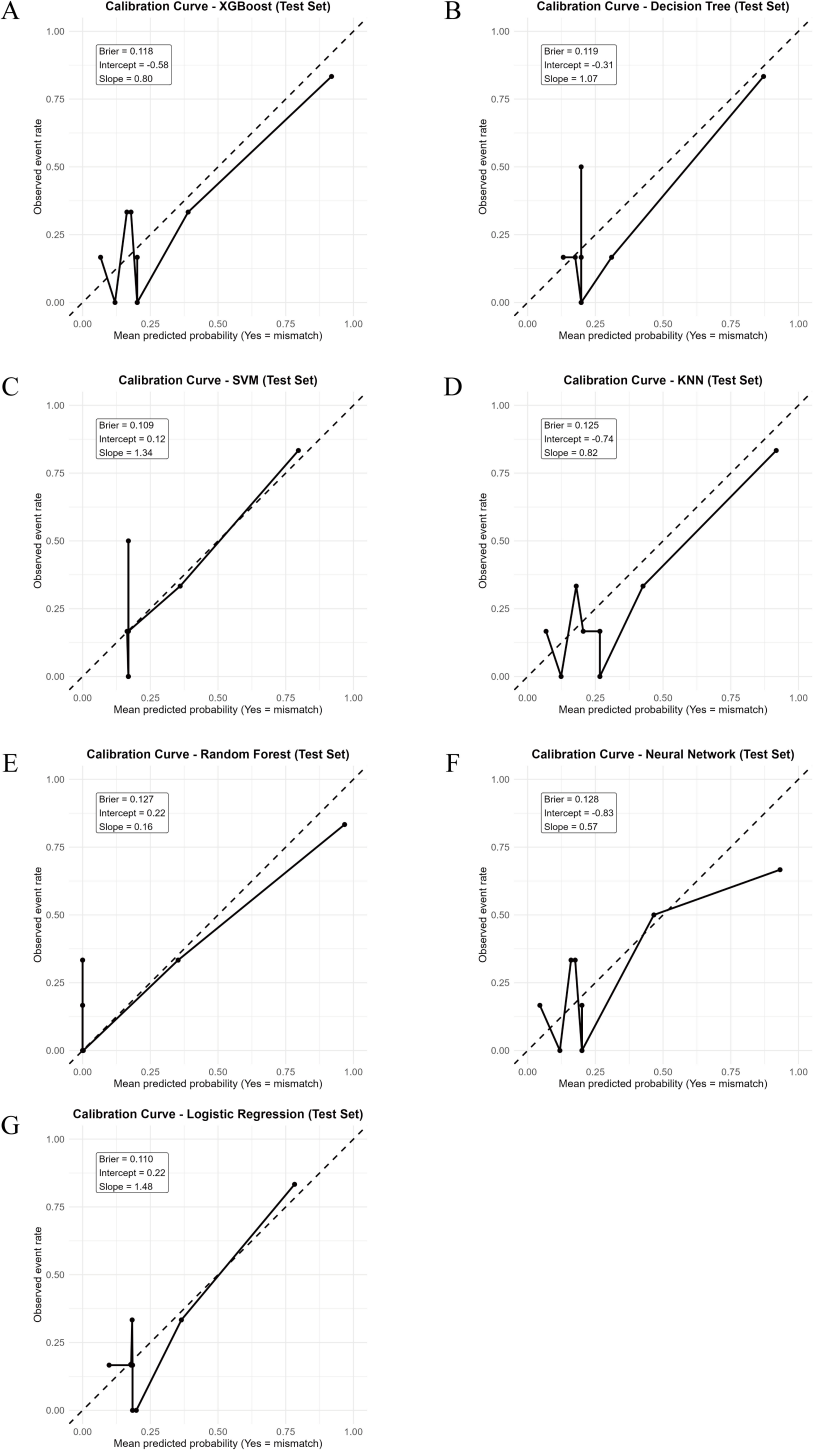
Calibration curves and calibration metrics of seven machine learning models in the testing dataset **(A–G)**. Calibration performance of the seven machine learning models in the testing dataset: **(A)** XGBoost (eXtreme Gradient Boosting), **(B)** decision tree, **(C)** support vector machine (SVM), **(D)** k-nearest neighbors (KNN), **(E)** random forest, **(F)** neural network, and **(G)** logistic regression. Each panel displays the calibration curve together with the Brier score, intercept, and slope, providing a quantitative assessment of model calibration. A curve closer to the diagonal reference line and calibration metrics approaching ideal values indicate better agreement between predicted and observed probabilities of EUS–pathology mismatch.

**FIGURE 5:**
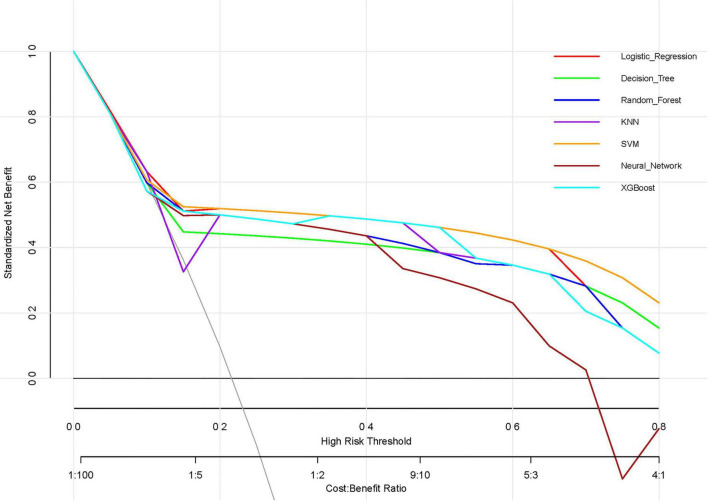
Decision curve analysis (DCA) of seven machine learning models in the testing dataset. Decision curve analysis comparing the net clinical benefit of the seven machine learning models across a range of threshold probabilities. The curves illustrate the clinical utility of each model in predicting EUS–pathology mismatch, with higher net benefit indicating better potential value for decision-making.

Comparison of F1-scores further supported the performance of SVM under class imbalance. SVM achieved the highest F1-score (0.667), reflecting a favorable balance between sensitivity and precision for detecting mismatch cases. Regarding calibration, SVM had the lowest Brier score in the test set (0.109) (Figure 4 and Table 3), suggesting minimal overall deviation between predicted probabilities and observed outcomes. Its calibration intercept was 0.12 and its calibration slope was 1.34, both close to ideal values. Decision curve analysis (Figure 5) further showed that SVM provided higher net benefit across a broader range of threshold probabilities.

In addition to conventional performance metrics, we examined the observed EUS–histopathology outcomes within the SVM-predicted groups in the test set. Using the Youden index-derived threshold determined in the training set, SVM classified 8 lesions as predicted mismatch and 52 lesions as predicted match. Among the 8 lesions in the predicted mismatch group, 7 were true mismatches, corresponding to an observed mismatch rate of 87.5%. Among the 52 lesions in the predicted match group, 46 were true matches, corresponding to an observed concordance rate of 88.5% (Table 4). These findings indicate that the SVM-predicted risk categories were associated with clinically meaningful differences in observed EUS–histopathology concordance.

**TABLE 4:** Observed EUS–histopathology outcomes according to SVM-predicted groups in the test set.

SVM-predicted group	Total, *n*	True match, *n* (%)	True mismatch, *n* (%)	Clinically relevant observed rate
Predicted match	52	46 (88.5%)	6 (11.5%)	Observed concordance rate: 88.5%
Predicted mismatch	8	1 (12.5%)	7 (87.5%)	Observed mismatch rate: 87.5%
Overall	60	47 (78.3%)	13 (21.7%)	Correctly classified: 53/60 (88.3%)

Taken together, SVM demonstrated the most robust overall performance in the independent test set across discrimination, classification metrics, calibration, and clinical net benefit. Therefore, SVM was selected for subsequent interpretability analysis and development of the online prediction platform.

As a robustness analysis, repeated stratified 10-fold cross-validation was further performed to reduce uncertainty associated with the relatively small hold-out test set. Across 100 validation folds, SVM achieved the highest cross-validated AUC among the candidate models, with an AUC of 0.713 (95% interval, 0.537–0.884), followed by random forest (0.711, 95% interval, 0.500–0.919), logistic regression (0.706, 95% interval, 0.499–0.942), XGBoost (0.697, 95% interval, 0.476–0.895), neural network (0.682, 95% interval, 0.468–0.901), KNN (0.676, 95% interval, 0.450–0.879), and decision tree (0.617, 95% interval, 0.404–0.713) (Supplementary Table 6). Although the cross-validated AUC estimate was more conservative than the hold-out test-set AUC, SVM remained the top-ranked model and showed performance comparable to random forest and logistic regression, supporting the robustness of final model selection.

### Model interpretation

To improve model transparency, SHapley Additive exPlanations (SHAP) were used to quantify the contribution of each predictor in the SVM model and provide both global and patient-level explanations.

**FIGURE 6:**
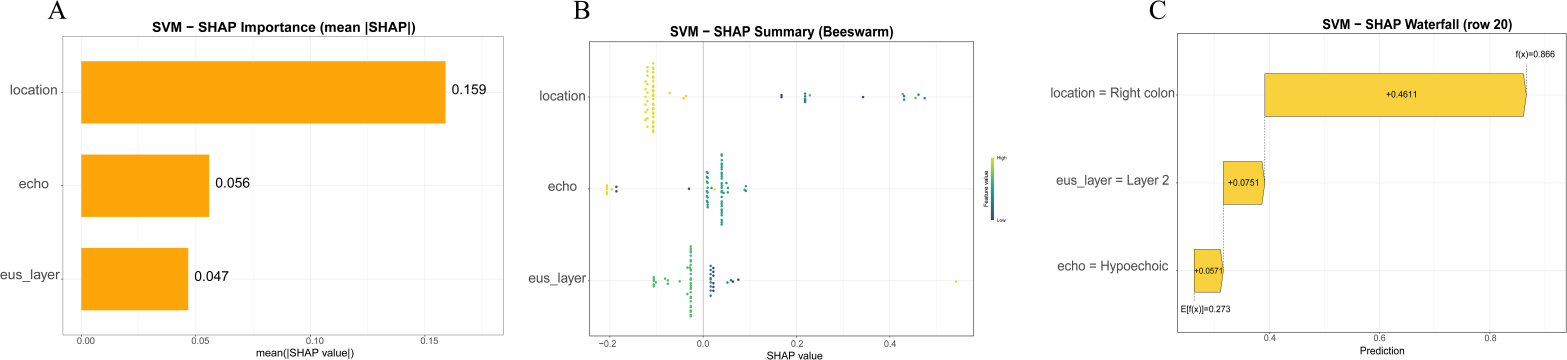
SHapley Additive exPlanations (SHAP)-based global and local interpretability of the SVM model **(A–C)**. **(A)** SHAP bar plot ranking features by mean absolute SHAP value, identifying location, echo, and eus_layer as the most influential predictors of EUS–histopathology mismatch.**(B)** SHAP beeswarm plot showing the distribution and direction of feature effects across cases; each dot represents one case, and positive SHAP values indicate contributions toward a higher predicted probability of mismatch. Lesion location shows the widest spread of SHAP values. Hypoechoic echogenicity generally shifts predictions toward higher mismatch risk, and deeper layers of origin tends to contribute positively in a subset of cases, whereas more superficial layers contribute less. **(C)** SHAP waterfall plot for a representative case (Patient 20), illustrating how features such as right colon location, layer 2 origin, and hypoechoic echogenicity shift the model output from the baseline toward a higher predicted mismatch risk, providing case-level interpretability.

Global interpretability summarized the overall decision logic of the model. In the SHAP bar plot (Figure 6A), features were ranked by their mean absolute SHAP values. Lesion location, echogenicity, and EUS layer of origin were the dominant predictors of EUS–histopathology mismatch risk, with mean absolute SHAP values of 0.159, 0.056, and 0.047, respectively. This indicates that lesion location played the leading role in model decision-making, while echogenicity and EUS layer of origin provided complementary information.

The SHAP beeswarm plot (Figure 6B) further showed that lesion location had the widest SHAP value distribution, indicating the greatest overall influence on model predictions. Stratified by anatomical site, rectal lesions generally had negative SHAP values, indicating a lower predicted risk of mismatch. In contrast, lesions located in the terminal ileum, left colon, and right colon tended to have positive SHAP values, indicating a higher predicted mismatch risk.

Echogenicity was the second most important feature. Hypoechoic lesions generally showed positive SHAP values, indicating an association with increased mismatch risk, whereas anechoic and hyperechoic lesions were more frequently located in the negative SHAP range and were therefore more likely to be predicted as concordant. The overall contribution of EUS layer of origin was smaller; however, some deeper-layer lesions, particularly those originating from layer 4, showed markedly positive SHAP values, suggesting that certain layers of origin may substantially increase mismatch risk.

**FIGURE 7:**
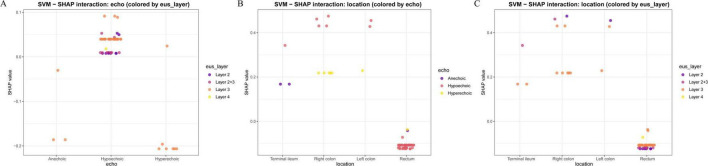
SHapley Additive exPlanations interaction plots illustrating pairwise feature interactions in the SVM model **(A–C)**. **(A)** Interaction between echo and eus_layer, showing that hypoechoic lesions consistently contribute positively to mismatch risk across layers, with the strongest effect observed in layer 3. **(B)** Interaction between location and echo, demonstrating that colonic lesions (left/right colon) exert a strong positive contribution to mismatch risk, particularly when combined with hypoechoic echogenicity, whereas rectal lesions show stable negative contributions. **(C)** Interaction between location and eus_layer, indicating that colonic lesions increase mismatch risk across different layers, with more pronounced effects in layer 2 and layer 2 + 3, while rectal lesions maintain a consistent protective effect across layers.

To explore interactions among predictors, SHAP interaction plots were generated for lesion location, echogenicity, and EUS layer of origin (Figure 7). The interaction between echogenicity and layer of origin (Figure 7A) suggested that hypoechoic echogenicity contributed positively to mismatch risk across layers, with the strongest trend observed for layer-3 lesions. The interaction between location and echogenicity (Figure 7B) showed that colonic locations, particularly the left and right colon, increased mismatch risk, especially in the presence of hypoechoic echogenicity. In contrast, rectal location consistently showed negative SHAP contributions, suggesting a relatively stable protective association. The interaction between location and layer of origin (Figure 7C) further demonstrated that colonic locations tended to increase mismatch risk across different layers, particularly for lesions originating from layer 2 or layer 2 + 3, whereas rectal lesions maintained negative contributions across layers.

Local interpretability illustrated how the model generated predictions for individual cases. For patient 20, the SHAP waterfall plot (Figure 6C) showed that a lesion located in the right colon, originating from layer 2, and exhibiting hypoechoic echogenicity all contributed positively to the predicted mismatch risk. The corresponding SHAP values were +0.4611, +0.0751, and +0.0571, respectively, indicating that these features jointly increased the model-estimated mismatch risk for this patient. The cumulative SHAP contributions in the waterfall plot demonstrate how the model integrates multiple clinical variables to generate an individualized prediction.

### Misclassification direction

To further characterize the major patterns of discordance between EUS and histopathological diagnoses, we constructed misclassification direction matrices based on the primary EUS diagnosis and the final histopathological diagnosis (Supplementary Tables 7, 8). When the primary EUS diagnosis was neuroendocrine tumor, most lesions were confirmed as neuroendocrine tumors on histopathology. However, a substantial proportion were ultimately classified as other pathological entities, including leiomyoma, chronic mucosal inflammation, rectal tonsil, granular cell tumor, and other low-frequency types. This finding suggests that, even when neuroendocrine tumor is assigned as the primary EUS diagnosis, the corresponding final pathological distribution remains heterogeneous.

In contrast, when the primary EUS diagnosis was lipoma or cyst, the final histopathological diagnosis was largely concordant with the EUS assessment, with relatively low misclassification rates. Lesions with a primary EUS diagnosis of GIST showed a more dispersed distribution on final pathology, with some lesions categorized as “Others” or confused with other rare entities.

Overall, certain high-frequency primary EUS diagnoses corresponded to heterogeneous final histopathological outcomes, whereas EUS diagnoses with more distinctive structural imaging features tended to map to a more concentrated pathological spectrum and were associated with lower misclassification rates.

### Implementation of the web-based calculator

**FIGURE 8:**
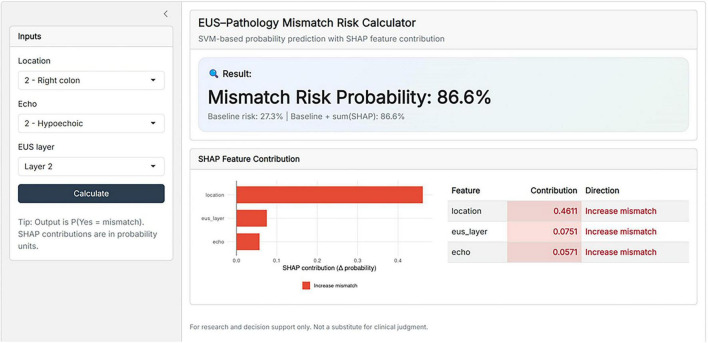
Web-based calculator integrating the SVM model to estimate the risk of mismatch between EUS assessment and pathological diagnosis in colorectal subepithelial lesions.

We integrated the final SVM model into an online, web-accessible risk calculator (Figure 8). Using the key predictors identified by LASSO and interpreted by SHAP, clinicians can enter relevant clinical and ultrasonographic parameters to obtain a real-time, individualized estimate of EUS–histopathology mismatch risk. To enhance transparency and interpretability, the platform also provides patient-level SHAP visualizations, including a ranked list of feature contributions and corresponding plots, thereby highlighting the main drivers of each prediction.

The web-based calculator can be accessed directly via the following link: https://runhua2025.shinyapps.io/eus_web_calculator-1/. It should be noted that the web tool is for research purposes only and cannot independently guide clinical care.

## Discussion

In this retrospective real-world cohort study, we developed and validated an interpretable machine-learning model to predict the risk of EUS–histopathology mismatch in colorectal SELs. Among seven candidate algorithms, the SVM model showed the most favorable overall performance in the independent test set. The final model relied on three routinely available EUS features—lesion location, echogenicity, and EUS layer of origin—and was further interpreted using SHAP. These findings suggest that a small set of structured EUS variables can provide useful information for identifying colorectal SELs in which EUS-based diagnosis may be less reliable.

Previous studies on diagnostic discordance between EUS and histopathology have several limitations when applied to colorectal SELs. Many have focused on upper gastrointestinal SELs (16), where anatomical conditions, pathological composition, and EUS operating environments differ substantially from those in the colorectum. Other studies have used a large number of predictors or complex model structures (17), which may limit clinical practicality. Models developed across the entire gastrointestinal tract may also overlook site-specific heterogeneity in colorectal lesions (18). In contrast, our study focused specifically on colorectal SELs and developed a parsimonious, interpretable, and web-deployable model based on variables routinely available after EUS examination.

In the head-to-head model comparison, SVM achieved the highest test-set AUC and showed balanced performance across accuracy, specificity, F1-score, calibration, and decision curve analysis. SVM may be particularly suitable for structured medical datasets with relatively low feature dimensionality, nonlinear relationships, and class imbalance because it identifies an optimal separating boundary in the feature space (19, 20). Prior studies have also supported the stability of SVM in medical prediction tasks based on structured clinical data (21). However, the higher AUC observed in the hold-out test set than in the training set should be interpreted cautiously. This finding is most likely attributable to sampling variability related to the relatively small test set, which included only 60 lesions and 13 mismatch events. If the held-out cases contained feature combinations that were more easily separable by the three selected predictors, the test-set AUC could appear higher than the training-set AUC. Consistently, repeated stratified 10-fold cross-validation yielded a more conservative SVM AUC of 0.713 (95% interval, 0.537–0.884), suggesting that the hold-out test-set AUC should not be overemphasized while still supporting the robustness of SVM as the final model.

SHapley Additive exPlanations analysis further clarified the decision logic of the final model. Lesion location was the most influential predictor of EUS–histopathology mismatch. Colonic lesions, particularly those in the left and right colon, were associated with higher mismatch risk, whereas rectal lesions showed a relatively protective pattern. This finding is clinically plausible because the colon is more tortuous, more mobile, and more affected by peristalsis, variations in lumen caliber, and residual gas, all of which can impair stable EUS imaging (22). These factors may lead to unstable ultrasound beam incidence, artifacts, and indistinct visualization of wall-layer boundaries, particularly outside the rectum (15, 23). In contrast, the rectum is relatively fixed and straighter, facilitating more stable imaging conditions. Moreover, the pathological composition of colonic SELs may be more heterogeneous (24), further increasing the likelihood of diagnostic overlap.

Echogenicity was another important contributor. Hypoechoic lesions were associated with increased mismatch risk, probably because hypoechogenicity is common but non-specific on EUS and may correspond to a broad range of pathological entities, including neuroendocrine tumors, low-risk GISTs, leiomyomas, lymphomas, and granular cell tumors (25). These lesions often share similar EUS appearances, such as hypoechoic texture, homogeneous or mildly heterogeneous internal echoes, and margins that may be well-defined or indistinct, making differentiation difficult based on echogenicity alone (9, 26). By contrast, anechoic or hyperechoic appearances often have clearer structural implications, such as cystic lesions or lipomas, and were associated with higher concordance (25).

Endoscopic ultrasonography layer of origin also contributed to mismatch risk. Lesions arising from deeper layers are more dependent on scanning angle, water-filling status, probe pressure, and image stability. When wall-layer boundaries are unclear or the bowel wall is compressed, the true layer of origin may be misclassified, which can subsequently bias pathological inference. In addition, several SEL entities may arise from adjacent layers or show trans-layer involvement, including leiomyoma, granular cell tumor, and lymphoma (5, 25), further weakening the specificity of the “layer–pathology” relationship. These findings support the clinical relevance of using location, echogenicity, and layer of origin together rather than relying on any single EUS feature.

The misclassification direction matrices provided additional insight into diagnostic discordance. Although neuroendocrine tumor was the most common primary EUS diagnosis and was frequently confirmed histologically, a subset of lesions assigned this diagnosis on EUS were ultimately classified as leiomyoma, chronic mucosal inflammation, rectal tonsil, granular cell tumor, or other less common entities. Similarly, lesions diagnosed as GIST on EUS showed a dispersed final pathological distribution. These findings suggest that certain high-frequency EUS diagnoses may function as a “diagnostic sink” when imaging features are insufficiently specific. In contrast, EUS diagnoses with more distinctive structural features, such as lipoma or cyst, were more consistently mapped to the corresponding histopathological categories. This pattern supports the positioning of our model: it is not intended to predict the exact pathological subtype, but to identify clinical contexts in which EUS-based diagnosis is more likely to deviate from histopathology.

The proposed model may have practical value as a post-EUS risk-alert tool. When the predicted risk of EUS–histopathology mismatch is high, clinicians may consider additional verification, such as tissue acquisition, repeat examination, expert review, or referral to a center with advanced EUS expertise, particularly when image quality is suboptimal or wall-layer boundaries are unclear. When the predicted risk is low, the EUS-based diagnosis may be considered more reliable, although management should still be individualized according to clinical context. Therefore, the model should support, rather than replace, endoscopists’ clinical judgment and should be interpreted as an auxiliary tool for identifying cases that may require more cautious evaluation.

Among operator- and examination-related variables, training status differed significantly between the match and mismatch groups, whereas physician seniority did not. This suggests that structured training may be more relevant than seniority alone for improving diagnostic concordance in colorectal SELs. The numerically higher proportion of senior physicians in the mismatch group should not be interpreted as evidence of inferior diagnostic capability. The percentages in Table 1 describe group composition rather than direct diagnostic performance, and senior physicians may be more likely to examine or review complex, technically challenging, or diagnostically uncertain cases. Moreover, physician type was not significantly associated with mismatch in baseline comparisons and was not retained during LASSO-based feature selection. In contrast, training status may influence diagnostic concordance by improving standardized scanning workflows, layer interpretation, and recognition of common pitfalls.

Notably, training status was not retained in the final model despite its association with mismatch in baseline analysis. This is not contradictory. LASSO identifies predictors with independent incremental value for outcome prediction, and the effect of training may be mediated through improved recognition of structured EUS features, such as lesion location, echogenicity, and layer of origin. Once these objective imaging features were included, training status may have provided limited additional predictive information. Excluding operator-dependent variables may also improve model stability and transportability across clinical settings.

This study has several strengths. First, it used real-world electronic medical record data, allowing the model to capture diagnostic uncertainty encountered in routine practice. Second, it focused specifically on colorectal SELs, a site-specific and technically challenging setting that differs from upper gastrointestinal SELs. Third, multiple machine-learning algorithms were compared within a unified framework incorporating independent testing, repeated cross-validation, calibration assessment, and decision curve analysis. Fourth, the final model was based on only three routinely available EUS features, facilitating clinical use. Finally, SHAP interpretation and web-based deployment improved model transparency and potential usability.

Several limitations should also be acknowledged. First, this was a retrospective single-center study, and selection bias is possible; therefore, generalizability may be limited across settings with different patient populations, equipment, and EUS practice patterns. Multicenter prospective validation is needed. Second, although electronic medical record data enhance clinical realism, retrospective datasets remain susceptible to unmeasured confounding, and future studies should use standardized prospective data collection. Third, although the sample size was adequate for model development and internal validation, the overall cohort remained modest, and additional external validation is required to confirm robustness and transportability.

In addition, the model predicts only the probability of EUS–histopathology mismatch and cannot determine the direction of misclassification, such as whether EUS underestimates a higher-risk lesion or overestimates a lower-risk lesion. This limitation may reduce its ability to distinguish clinically different consequences of mismatch. Therefore, the model should be used as a risk-alert tool rather than a tool for determining the specific severity or direction of diagnostic error.

Another limitation is that the model was built on structured EUS features rather than raw images and therefore may not capture potentially informative image-level signals. Future studies could integrate image-based approaches with structured variables to improve predictive performance while preserving interpretability. Moreover, the three core predictors—lesion location, echogenicity, and EUS layer of origin—are routinely available but still partly operator-dependent. Echogenicity and layer of origin may be particularly susceptible to interobserver variability because they depend on image quality, scanning angle, bowel preparation, probe stability, and individual interpretive experience. Therefore, the reliability of the web-based calculator depends on the consistency and accuracy of these input variables. To improve reproducibility, the calculator should be used together with standardized EUS reporting, structured training, and careful review of uncertain cases. It should be regarded as a post-EUS risk-alert tool rather than an independent diagnostic system.

Finally, although operator-related factors such as training status were associated with mismatch in baseline analysis, they were not retained in the final model. This may limit representation of certain process-related determinants of diagnosis. Future prospective studies incorporating standardized training pathways could more systematically evaluate how training influences image quality, feature interpretation, and EUS–histopathology concordance.

## Conclusion

In this retrospective study, we developed and validated an interpretable machine-learning model to estimate the risk of EUS–histopathology mismatch in colorectal SELs using routinely available EUS features. The final SVM model, based on lesion location, echogenicity, and EUS layer of origin, demonstrated favorable predictive performance and interpretability. SHAP analysis provided transparent explanations of feature contributions, and the web-based calculator enabled individualized risk estimation. Future multicenter prospective studies are needed to externally validate this model and evaluate its potential role as a post-EUS risk-alert tool in clinical practice.

## Data Availability

The raw data supporting the conclusions of this article will be made available by the authors, without undue reservation.
