# Supplementary material for: Development and validation of an interpretable predictive model for mismatch between endoscopic ultrasonography and histopathological diagnosis in colorectal subepithelial lesions: a retrospective single-center study
Source: Front Med (Lausanne). 2026 Jul 30;13:1831811. doi: 10.3389/fmed.2026.1831811 (PMC13467990; doi:10.3389/fmed.2026.1831811)
Supplement: Supplementary file 1 [file Table_1.docx]

**Supplementary Table 1 Information description of included variables**

| **Variable** | **Variable type** | **Categories/ scale** | **Description** |
| --- | --- | --- | --- |
| **Sex (sex)** | Categorical | Male; Female | Patient sex. |
| **Age (age)** | Continuous | Years | Age at EUS examination (years). |
| **Lesion location (location)** | Categorical | Terminal ileum; Right colon; Left colon; Rectum | Anatomical location of the lesion. |
| **maximum lesion diameter (size_mm)** | Continuous | mm | Maximum lesion diameter measured on EUS (mm). |
| **Echogenicity (echo)** | Categorical | Anechoic; Hypoechoic; Hyperechoic | Echogenicity of the lesion on EUS. |
| **EUS layer of origin (eus_layer)** | Categorical | Layer 2; Layer 2+3; Layer 3; Layer 4 | Layer of origin as assessed by the endoscopist on EUS. “Layer 2+3” indicates involvement of both the second and third layers. |
| **Image quality (img_q)** | Categorical | Low; Medium; High | EUS image quality was assessed based on a modified scoring system adapted from Yamamoto et al.[1] using three items: (1) appropriate distance (the probe should not compress the lesion), (2) clear structure (the lesion and adjacent wall layers are clearly visualized), and (3) clear background (>50% of the background without obvious artifacts). Each satisfied item scored 1 point (total 0–3). Scores 0–1 were defined as low quality, 2 as medium quality, and 3 as high quality. |
| **Physician type (doc_type)** | Categorical | Junior physician; Senior physician | Senior physicians were defined as those having performed >200 EUS examinations; junior physicians ≤200. |
| **Pre-/post-training status (training)** | Categorical | Pre-training; Post-training | A subset of physicians attended a structured EUS diagnostic training session on SELs on March 11, 2021; these physicians were classified as post-training, whereas others were classified as pre-training. |
| **EUS modality (eus_type)** | Categorical | Radial; Linear; Miniprobe | Type of EUS device used. Conventional EUS included radial (EG-580UR, EG-530UR2; Fujifilm, Japan) and linear (UCT260; Olympus, Japan; EG-580UT, EG-530UT2; Fujifilm, Japan). Miniprobes included IM-02M-01 (Yingmeida, China), UM-2R and UM-3R (Olympus, Japan). Ultrasound processors included ME2-Plus (Olympus, Japan), SU9000 (Fujifilm, Japan), and IM-02M-01 (Yingmeida, China). |
| **Outcome label (label)** | Binary outcome variable | Match; Mismatch | Concordance between the primary EUS diagnosis and the final histopathological diagnosis. Mismatch was defined as discordance at the diagnostic category/specific pathological type level; differential diagnoses listed in the EUS report were not counted as “match”. |

1.	Yamamoto S, Nishida T, Kato M, Inoue T, Hayashi Y, Kondo J, Akasaka T, Yamada T, Shinzaki S, Iijima H, Tsujii M, Takehara T (2012) Evaluation of endoscopic ultrasound image quality is necessary in endosonographic assessment of early gastric cancer invasion depth. Gastroenterol Res Pract 2012:1–5. https://doi.org/10.1155/2012/194530

**Supplementary Table 2. Match rate stratified by final histopathological diagnosis (top categories and Others)**

| **Final histopathological diagnosis** | **Total (n)** | **Match (n)** | **Mismatch (n)** | **Match rate (%)** |
| --- | --- | --- | --- | --- |
| **Neuroendocrine tumor** | 169 | 168 | 1 | 99.4% |
| **Lipoma** | 35 | 32 | 3 | 91.4% |
| **Others** | 29 | 6 | 23 | 20.7% |
| **Leiomyoma** | 17 | 2 | 15 | 11.8% |
| **Rectal tonsil** | 13 | 2 | 11 | 15.4% |
| **Chronic mucosal inflammation** | 12 | 0 | 12 | 0.0% |
| **Granular cell tumor** | 10 | 1 | 9 | 10.0% |
| **Cyst** | 9 | 9 | 0 | 100.0% |
| **Fibrous hyperplasia** | 7 | 2 | 5 | 28.6% |

**Supplementary Table 3. Clinical scenario–stratified concordance between EUS and histopathology**

| **Variable** | **Category** | **Total, n** | **Match, n** | | **Mismatch, n** | | **Concordance rate, %** | **Mismatch rate, %** |
| --- | --- | --- | --- | --- | --- | --- | --- | --- |
| Overall | — | 301 | 222 | 79 | | 73.8 | | 26.2 |
| Sex | Female | 123 | 88 | 35 | | 71.5 | | 28.5 |
|  | Male | 178 | 134 | 44 | | 75.3 | | 24.7 |
| Lesion location | Terminal ileum | 11 | 7 | 4 | | 63.6 | | 36.4 |
|  | Right colon | 43 | 26 | 17 | | 60.5 | | 39.5 |
|  | Left colon | 30 | 12 | 18 | | 40.0 | | 60.0 |
|  | Rectum | 217 | 177 | 40 | | 81.6 | | 18.4 |
| Echogenicity | Anechoic | 12 | 11 | 1 | | 91.7 | | 8.3 |
|  | Hypoechoic | 245 | 174 | 71 | | 71.0 | | 29.0 |
|  | Hyperechoic | 44 | 37 | 7 | | 84.1 | | 15.9 |
| EUS layer of origin | Layer 2 | 52 | 34 | 18 | | 65.4 | | 34.6 |
|  | Layer 2+3 | 54 | 41 | 13 | | 75.9 | | 24.1 |
|  | Layer 3 | 184 | 144 | 40 | | 78.3 | | 21.7 |
|  | Layer 4 | 11 | 3 | 8 | | 27.3 | | 72.7 |
| Image quality | Low | 46 | 28 | 18 | | 60.9 | | 39.1 |
|  | Medium | 110 | 87 | 23 | | 79.1 | | 20.9 |
|  | High | 145 | 107 | 38 | | 73.8 | | 26.2 |
| Physician type | Junior | 95 | 75 | 20 | | 78.9 | | 21.1 |
|  | Senior | 206 | 147 | 59 | | 71.4 | | 28.6 |
| Training status | Pre-training | 126 | 84 | 42 | | 66.7 | | 33.3 |
|  | Post-training | 175 | 138 | 37 | | 78.9 | | 21.1 |
| EUS modality | Radial | 226 | 160 | 66 | | 70.8 | | 29.2 |
|  | Linear | 6 | 6 | 0 | | 100.0 | | 0.0 |
|  | Mini-probe | 69 | 56 | 13 | | 81.2 | | 18.8 |

**Supplementary Table 4. Optimal hyperparameters for each algorithm (determined within the training set) and tuning strategies**

| **Algorithm** | **Optimal hyperparameters used** | **Tuning strategy** |
| --- | --- | --- |
| **Logistic Regression** | Binomial logit model (no tunable hyperparameters) | Not applicable |
| **KNN** | k=26；kernel=triangular；distance=2 | 5-fold CV in the training set; k selected to maximize AUC (kernel and distance fixed) |
| **Decision tree** | cp=0.016 | 10-fold CV grid search in the training set; cp selected to maximize AUC |
| **Random Forest** | mtry=3；ntree=600 | mtry tuned via CV in the training set; ntree selected by minimizing OOB error in the training set by scanning ntree = 50–1000 (step = 50) (set.seed = 111) |
| **SVM** | cost = 10; gamma = 0.01; class weights (set proportional to the match/mismatch class distribution) | 10-fold CV grid search in the training set; cost and gamma selected to maximize AUC; class weights applied to mitigate class imbalance |
| **Neural Network** | hidden = 3 (single hidden layer with 3 units) | 5-fold CV in the training set; network size (hidden units) selected to maximize AUC |
| **XGBoost** | objective=binary:logistic；eval_metric=auc；max_depth=5；eta=0.2；subsample=0.8；colsample_bytree=1；nrounds=21 | 5-fold (stratified) CV with grid search in the training set; parameters selected to maximize mean test AUC. Maximum nrounds set to 1000 with early stopping (30 rounds); final nrounds determined by best_iteration (= 21) |

**Notes:** All hyperparameters were tuned exclusively within the training set; the test set was used only for final independent evaluation.
**Abbreviations:** AUC, area under the receiver operating characteristic curve; CV, cross-validation; KNN, k-nearest neighbors; OOB, out-of-bag; XGBoost, eXtreme Gradient Boosting; SVM, support vector machine.

**Supplementary Table 5. Performance of each algorithm in the training set**

| **Model** | **AUC** | **Accuracy** | **Sensitivity** | **Specificity** | **PPV** | **NPV** | **F1-score** | **Brier score** | **Threshold_Youden** |
| --- | --- | --- | --- | --- | --- | --- | --- | --- | --- |
| **KNN** | 0.701 | 0.838 | 0.515 | 0.960 | 0.829 | 0.840 | 0.636 | 0.134 | 0.350 |
| **Random Forest** | 0.736 | 0.838 | 0.515 | 0.960 | 0.829 | 0.840 | 0.636 | 0.155 | 0.322 |
| **Neural Network** | 0.792 | 0.838 | 0.515 | 0.960 | 0.829 | 0.840 | 0.636 | 0.129 | 0.395 |
| **Logistic Regression** | 0.748 | 0.830 | 0.485 | 0.960 | 0.821 | 0.832 | 0.610 | 0.143 | 0.256 |
| **Decision Tree** | 0.712 | 0.822 | 0.409 | 0.977 | 0.871 | 0.814 | 0.557 | 0.146 | 0.534 |
| **XGBoost** | 0.790 | 0.838 | 0.515 | 0.960 | 0.829 | 0.840 | 0.636 | 0.131 | 0.318 |
| **SVM** | 0.789 | 0.834 | 0.500 | 0.960 | 0.825 | 0.836 | 0.623 | 0.138 | 0.485 |

**Abbreviations:** AUC, area under the receiver operating characteristic curve; PPV, positive predictive value; NPV, negative predictive value; KNN, k-nearest neighbors; XGBoost, eXtreme Gradient Boosting; SVM, support vector machine.

**Supplementary Table 6. Cross-validated AUCs of machine-learning models**

| **Model** | **N resamples** | **Cross-validated AUC (95% interval)** |
| --- | --- | --- |
| **SVM** | 100 | 0.713 (0.537–0.884) |
| **Random Forest** | 100 | 0.711 (0.500–0.919) |
| **Logistic Regression** | 100 | 0.706 (0.499–0.942) |
| **XGBoost** | 100 | 0.697 (0.476–0.895) |
| **Neural Network** | 100 | 0.682 (0.468–0.901) |
| **KNN** | 100 | 0.676 (0.450–0.879) |
| **Decision Tree** | 100 | 0.617 (0.404–0.713) |

**Abbreviations:** AUC, area under the receiver operating characteristic curve; KNN, k-nearest neighbors; SVM, support vector machine; XGBoost, eXtreme Gradient Boosting.
**Note:** Repeated stratified 10-fold cross-validation was performed 10 times, yielding 100 validation folds. The 95% interval represents the empirical interval across repeated validation folds.

**Supplementary Table 7. Misclassification direction matrix (counts): primary EUS diagnosis (rows) versus final histopathological diagnosis (columns) (n = 301 lesions)**

| **Primary EUS diagnosis** | **Others** | **Rectal tonsil** | **Neuroendocrine tumor** | **Granular cell tumor** | **Cyst** | **Leiomyoma** | **Chronic mucosal inflammation** | **Fibrous hyperplasia** | **Lipoma** |
| --- | --- | --- | --- | --- | --- | --- | --- | --- | --- |
| **Neuroendocrine tumor** | 17 | 8 | 168 | 6 | 0 | 13 | 11 | 3 | 3 |
| **Lipoma** | 0 | 2 | 0 | 0 | 0 | 1 | 0 | 0 | 32 |
| **Cyst** | 1 | 0 | 0 | 0 | 9 | 0 | 0 | 0 | 0 |
| **Gastrointestinal stromal tumor (GIST)** | 6 | 0 | 0 | 1 | 0 | 1 | 0 | 1 | 0 |
| **Others** | 1 | 2 | 1 | 1 | 0 | 0 | 0 | 0 | 0 |
| **Leiomyoma** | 0 | 1 | 0 | 1 | 0 | 2 | 0 | 0 | 0 |
| **Intestinal schistosomiasis** | 1 | 0 | 0 | 1 | 0 | 0 | 1 | 1 | 0 |
| **Fibrous hyperplasia** | 1 | 0 | 0 | 0 | 0 | 0 | 0 | 2 | 0 |
| **Lymphangioma** | 2 | 0 | 0 | 0 | 0 | 0 | 0 | 0 | 0 |

**Supplementary Table 8. Misclassification direction matrix (row percentages): primary EUS diagnosis (rows) versus final histopathological diagnosis (columns) (each row sums to 100%)**

| **Primary EUS diagnosis** | **Others** | **Rectal tonsil** | **Neuroendocrine tumor** | **Granular cell tumor** | **Cyst** | **Leiomyoma** | **Chronic mucosal inflammation** | **Fibrous hyperplasia** | **Lipoma** |
| --- | --- | --- | --- | --- | --- | --- | --- | --- | --- |
| **Others** | 20.0% | 40.0% | 20.0% | 20.0% | 0.0% | 0.0% | 0.0% | 0.0% | 0.0% |
| **Cyst** | 10.0% | 0.0% | 0.0% | 0.0% | 90.0% | 0.0% | 0.0% | 0.0% | 0.0% |
| **Leiomyoma** | 0.0% | 25.0% | 0.0% | 25.0% | 0.0% | 50.0% | 0.0% | 0.0% | 0.0% |
| **Lymphangioma** | 100.0% | 0.0% | 0.0% | 0.0% | 0.0% | 0.0% | 0.0% | 0.0% | 0.0% |
| **Neuroendocrine tumor** | 7.4% | 3.5% | 73.4% | 2.6% | 0.0% | 5.7% | 4.8% | 1.3% | 1.3% |
| **Fibrous hyperplasia** | 33.3% | 0.0% | 0.0% | 0.0% | 0.0% | 0.0% | 0.0% | 66.7% | 0.0% |
| **Intestinal schistosomiasis** | 25.0% | 0.0% | 0.0% | 25.0% | 0.0% | 0.0% | 25.0% | 25.0% | 0.0% |
| **Gastrointestinal stromal tumor (GIST)** | 66.7% | 0.0% | 0.0% | 11.1% | 0.0% | 11.1% | 0.0% | 11.1% | 0.0% |
| **Lipoma** | 0.0% | 5.7% | 0.0% | 0.0% | 0.0% | 2.9% | 0.0% | 0.0% | 91.4% |
